# Dysfunction of the Prefrontal Cortex in Betel-Quid–Dependent Chewers

**DOI:** 10.3389/fpsyt.2020.558367

**Published:** 2020-09-24

**Authors:** Lingyu Kong, Chang Zeng, Fulai Yuan, Shaohui Liu, Dongcui Wang, Canhua Jiang, Zhongyuan Zhan, Zhaoxin Qian, Xueling Zhu

**Affiliations:** ^1^Department of Radiology, Xiangya Hospital, Central South University, Changsha, China; ^2^Health Management Center, Xiangya Hospital, Central South University, Changsha, China; ^3^Department of Oral and Maxillofacial Surgery, Xiangya Hospital, Central South University, Changsha, China; ^4^Department of Emergency, Xiangya Hospital, Central South University, Changsha, China

**Keywords:** betel quid dependence, brain function, prefrontal cortex, cue reactivity task, go/nogo task

## Abstract

Betel quid is the fourth most popular psychoactive agent worldwide. Imaging studies have found altered brain structure in prefrontal cortex (PFC) in betel-quid dependent (BQD) chewers. However, the brain function in PFC associated with BQ use still remains unclear. The present study aimed to examine brain functional activity in PFC in individuals with BQD. This study recruited 48 participants with BQD and 22 normal controls (NCs). Both BQ-specific cue reactivity and Go/NoGo tasks were administered with functional magnetic resonance imaging (fMRI). Behavioral results showed a deficit in the choice reaction time task in BQD group. The fMRI results of the cue reactivity task suggested that, individuals with BQD exhibited responses in right ventromedial PFC, left posterior cingulate cortex (PCC), left lateral parietal lobe (LPL), left middle temporal gyrus and left visual cortex, when seeing BQ images compared with control images. In the Go/NoGo task, relative to NCs group, individuals with BQD showed higher activity in right dorsolateral PFC, right PCC and bilateral LPL between NoGo and Go trials. Across these two tasks, we consistently found disrupted function in PFC in individuals with BQD, which might lead to impaired craving and response inhibition in BQ addiction. Results of current study might shed light on the neural mechanisms involved in BQ use, which could be used as potential guidelines for diagnosis and treatment of BQ dependence.

## Introduction

Areca nut, the fruit of the areca palm, contains the principal active agent Arecoline (1), which is demonstrated to have a chemical structure analogous to that of nicotine (2). Thus, betel quid (BQ, the product of areca nut) is recognized as a group 1 carcinogen by International Agency for Cancer Research (1), and categorized as “addiction” when heavy use according to DSM-IV and ICD-10 (3, 4). BQ is now the fourth most commonly consumed psychoactive substance in the world, following only alcohol, nicotine, and caffeine (5, 6). A recent study with a large sample size in 6 Asian populations revealed a vast prevalence of BQ use in these counties (typically over 10%) (7). BQ use is reported to be highly associated with oral sub-mucous fibrosis and oral cancer (8–11). However, the limited understanding of BQ use makes it difficult for the development of effective treatment to those patients (12).

Several lines of researches have suggested that intensive BQ use shows similar symptoms of other addictive disorders. First, in the Asian Betel-quid Consortium study of 8922 participants from 6 countries, betel-quid use disorder was reported to meet DSM-V criteria for substance use disorder and had a high prevalence among users of betel-quid (7). Another study suggested that the 12-month prevalence of BQ use disorder was 18.0%, which exceeded that reported for DSM-V defined drug use disorder (3.9%) (13) and was comparable to the results of alcohol (13.9%) and nicotine (20.0%) use disorders (14, 15). Second, animal studies suggested that high dose of BQ use could induce cocaine-like physiological states, such as anxiety, dilated pupils, tachycardia, and elevated blood pressure (16). In addition, BQ use was reported to be associated with altered cognitive ability, including spatial short-term memory (17), attention and inhibition control (18), executive function (19) and attention bias to BQ cues (20).

Functional neuroimaging studies have implicated that structural and functional alterations related to BQ chewing and dependence. Recently, a review systematically assessed previous functional magnetic resonance imaging (fMRI) studies on BQ addiction, and suggested that a key involvement of the prefrontal cortex (PFC) in BQ dependency (21). For example, studies from our group suggested that BQ use was associated with decreased gray matter volumes in bilateral dorsolateral PFC and ventral medial PFC (22), altered white matter integrity in anterior thalamic radiation highly associated with PFC (23), and less cortical thickness in PFC (19). These neuroimaging studies proposed that the role of prefrontal cortical structures in BQ addiction, which plays an important role in cognitive behavioral and emotional changes in various kinds of addiction (24–27). However, all the evidence in BQ addiction mainly came from studies of brain structure in BQ users. Little is known about functional role of PFC in individuals with betel-quid dependence (BQD).

Two common tasks employed in addiction neuroimaging studies are the cue reactivity and Go/NoGo tasks, in which neural activation is measured while the subject is viewing neutral versus addiction-related images and conducting the addiction-related Go and NoGo stimulus. Both of them can evoke the response from PFC. The present study aimed to investigate neural activity in PFC in individuals with BQD compared to normal controls (NCs). To do so, 48 men with BQD and 22 NCs were recruited to perform the BQ-related cue reactivity and Go/NoGo tasks under fMRI investigation. Consistent with previous findings that BQ use may be associated in part with structural deficits in PFC, we hypothesized that abnormal activation in PFC in individuals with BQD in both BQ-related cue reactivity and Go/NoGo tasks. We hoped that current study could improve our understanding of the roles of the PFC in BQ use.

## Materials and Methods

### Participants

Seventy male volunteers were recruited to participant in this study including 48 subjects with BQD and 22 NCs. Individuals with BQD met the following inclusion criteria: (1) 18 to 50 years of age; (2) Han nationality; (3) right-handed; (4) nine or more years of education; (5) meeting the diagnostic criteria for BQD according to DSM-V criteria for substance use disorders; (6) with no attempt to quit or experience of BQ abstinence in the past 6 months. The BQ use behaviors of BQD participants were recorded, including duration of BQ use (years), age of first BQ use, daily weight of BQ use (g), the number of BQ use days in a week and the brands of BQ products. Body mass index (BMI) was also recorded. The severity of BQ dependence was measured by Betel Quid Dependence Scale (BQDS) (28). The BQD group was additionally screened for alcohol and nicotine addiction with Alcohol Use Disorders Identification Test (AUDIT) and Fagerstrom Test for Nicotine Dependence (FTND).

Healthy subjects with matched age without BQ use were defined as the NCs group, which were recruited through a combination of targeted site sampling, advertisement and snowball sampling referrals. Subjects were excluded both as BQD chewers and healthy controls if they: (1) met the DSM-V criteria for other substance dependency, such as alcohol and nicotine; (2) had medical history of any neurological or psychiatric disorder; (3) had systemic illnesses (e.g. diabetes mellitus, cardiovascular disease, thyroid disorders, renal disorders, and epilepsy); (4) had contraindication to MRI examination; (5) had current use of psychotropic medications; (6) were not able to read and write Chinese or left–handed. This study was carried out in accordance with the recommendations of the research ethical committee of Xiangya Hospital of Central South University of Hunan Province, Changsha, China. All subjects were given written informed consent in accordance with the Declaration of Helsinki. The protocol was approved by the Institutional Review Board at Xiangya Hospital of Central South University of Hunan Province, Changsha, China.

### Behavior Interviews and Cognitive Tests

Participants came to Xiangya Hospital to finish behavior interview and MRI scans in the same day. The behavior interview included several parts. The first part was the basic demographic information collection, including age, years of education, BMI, BQ use behaviors and so on. Then, they were asked to complete three simple cognitive tasks: choice reaction time task, working memory task, as well as the classic color-word Stroop task.

The choice reaction time task was adapted with default settings from Deary, Liewald and Nissan (29). The time required to finish this task was used as the index of general reaction time measure. The two-back working memory task with numbers was adapted from Xue and colleagues (30) and the number of correct response in each block was served as the index of working memory. The Chinese adaptation of Stroop task was used to assess the inhibition ability. The number of correct responses in 45s in the Color-Word condition was recorded as the index for this task.

### MRI Protocol

In order to reduce the influence of addictive substances, all participants were required not to use caffeine, nicotine, alcohol and other addictive substance on the day of scanning. All MRI images were acquired using a Siemens 3.0T Prisma scanner at Xiangya Hospital. Standard settings were used to perform the scan. For example, foam pads were used to minimize head motion. Participants were instructed to have a rest, kept their head very still during the structural scan and responded to the instructions when doing functional scans. Stimulus presentation and timing of all stimuli and response events were achieved using Matlab (Mathworks) and Psychtoolbox (www.psychtoolbox.org) on an IBM-compatible PC. Participants’ responses were collected online using an MRI-compatible button box. The structural scan was performed using T1 MPRAGE sequence, covering whole brain with the following scanning parameters: TR/TE = 1900/3.18 ms, matrix = 320 × 320, number of slices = 256 and voxel size = 0.73 × 0.73 × 0.73 mm^3^, sagittal slice position. Functional scans were performed using EPI sequence with the following parameters: TR/TE = 2000 ms/30 ms, Matrix = 94 × 94, number of axial slices = 75, voxel size = 2.34 × 2.34 × 2.00 mm^3^.

### fMRI Tasks

Participants performed one session of cue reactivity task and two sessions of Go/NoGo task inside the scanner. During the session of cue reactivity task, two types of cues were presented: the BQ image and control images. There were 20 images for each category, and each image were presented for three times. In order to keep the participants awake during this passive view task, 10 animal images were presented twice. These images were presented with a random order with each image was presented for 3 s. Participants were instructed to press a button whenever they saw an animal. There were two sessions of Go/NoGo tasks: (1) a control Go/BQ NoGo task (CGo task) in which they were asked to press a button when they saw a control image, and refrained from pressing the button when they saw a BQ-related image, and (2) a BQ Go/control NoGo task (BGo task) in which they were asked to press a button when they saw a BQ-related image, and refrained from pressing the button when they saw a control image. This Go/NoGo paradigm was adapted from previous studies (31), and allowed an examination of brain responses to BQ stimuli and the inhibition of pre-potent responses to BQ stimuli. Consistent with previous studies (31), each session consisted of 120 Go trials (75%) and 40 NoGo trials (25%). The NoGo trials were presented in pseudo-randomized order so that NoGo trial appeared with equal probability after one to five consecutive Go trials, and two NoGo trials appeared consecutively. Each stimulus was presented for 500 ms, followed by a fixation cross for 1.5 to 4 s with a mean of 2.5 s. The order of two versions of GO-NoGO tasks was counterbalanced across participants.

The correct ratio and reaction in the cue task were served as the behavior data of the task. The correct ratio for Go and NoGo trials as well as the reaction time for Go trials for each session were calculated as indexes for habitual-impulsive responding to the stimuli and inhibition of NoGo stimuli. Then, these behavior data were compared across groups.

### fMRI Data Analysis

Image preprocessing and statistical analysis were carried out using FSL (www.fmrib.ox.ac.uk/fsl). Images were realigned to compensate for small residual head movements (32). Translational movement parameters never exceeded one voxel in any direction for any participant. Data was spatially smoothed using a 5-mm full-width-half-maximum (FWHM) Gaussian kernel and filtered using a nonlinear high pass filter with a 100-s cutoff.

A two-step registration procedure was used where EPI images were first registered to the MPRAGE structural image, and then into standard MNI space, using affine transformations (32). Registration from MPRAGE structural image to standard space was further refined using FNIRT nonlinear registration (33, 34). Statistical analyses were performed in the native image space with statistical maps normalized to the standard space prior to higher-level analyses. The data was modeled at the first-level using a general linear model within FSL’s FILM module.

For the cue task, brain activations were modeled for BQ images, control images, and animal images separately. For the Go/NoGo task, brain activation in every trial was modeled for BQ Go, BQ Nogo, control Go, and control NoGo trials respectively. Error-related trials (misses and false alarms) were modeled together as a nuisance variable. The event onsets were convolved with canonical hemodynamic response function (HRF, double-gamma function) to generate regressors. Temporal derivatives were included as covariates of no interest in order to improve statistical sensitivity. Null events were not explicitly modeled, and therefore constituted an implicit baseline. The six movement parameters were also included as covariates in the model.

Higher-level analysis was created to model the activity difference across two groups. For cue reactivity task, the activation difference of BQ images vs. control images was compared between two groups to reveal the interaction between stimuli and group. For the Go/NoGo tasks in both sessions, the difference of NoGo trials vs. Go trials was compared to reveal the interaction between task and group. Higher-level random-effect models were tested for group analyses using FMRIB’s Local Analysis of Mixed Effect stage 1 only (35, 36) with automatic outlier detection (37). The brain activation difference of BQ and control images, as well as NoGo and Go trials was also examined for BQD and NCs group independently using one-sample t-tests. Result images were thresholded with a height threshold of Z > 3.1 and a cluster probability of p < 0.05, corrected for whole-brain multiple comparisons based on Gaussian random field theory. The years of education was included as a covariate for all fMRI analysis.

## Results

### Behavioral Results

**Table 1** showed the demographic and cognitive characteristics for all participants. These two groups were matched on age and BMI, but they showed differences in years of education. In BQD group, they showed an average of 15.23 years of using BQ, average age 17.13 to try their first BQ and 40.19 grams of BQ per day. In terms of cognitive tests, these two groups showed no difference regarding the Stroop task as well as the working memory task. Compared with NCs group, BQD group showed deficits in the choice reaction task (**Table 1**). Correlation analysis showed that BQDS score was significantly correlated with the following variables in BQD group: (1) years of education, *r*(48) = −0.338, *p* = 0.019; (2) estimated daily use in gram, *r*(48) = 0.461, *p* < 0.001. These results suggested that participants with higher BQDS scores were those with less education and higher daily consumption.

**Table 1:** Demographic and clinical/cognitive characteristics of participants (M ± SD).

	BQD	NCs	Statistics
N	48	22	-
Age (years)	34.85 ± 8.10	32.05 ± 6.25	*t*(68) = 1.44, *p* = 0.15
Education (years)	11.63 ± 2.83	17.82 ± 2.82	*t*(68) = −6.19, *p* < 0.001
BMI (kg/m^2^)	25.07 ± 3.74	23.32 ± 3.84	*t*(68) = 1.81, *p* = 0.08
BQDS	59.63 ± 14.55	–	–
AUDIT	4.35 ± 3.68	–	–
FTND	3.73 ± 2.79	–	–
Duration of BQ use (years)	15.23 ± 7.10	–	–
Age of first BQ use	17.13 ± 6.67	–	–
Daily weight of BQ use (g)	40.19 ± 33.11	–	–
Choice Reaction Time (s)	35.21 ± 9.77	41.50 ± 10.29	*t*(68) = −2.46, *p* = 0.02
Stroop	78.50 ± 32.49	69.72 ± 24.72	*t*(68) = 1.13, *p* = 0.26
Working Memory	9.19 ± 1.14	9.50 ± 1.01	*t*(68) = −1.10, *p* = 0.28

### Cue Reactivity Task

**Figure 1:**
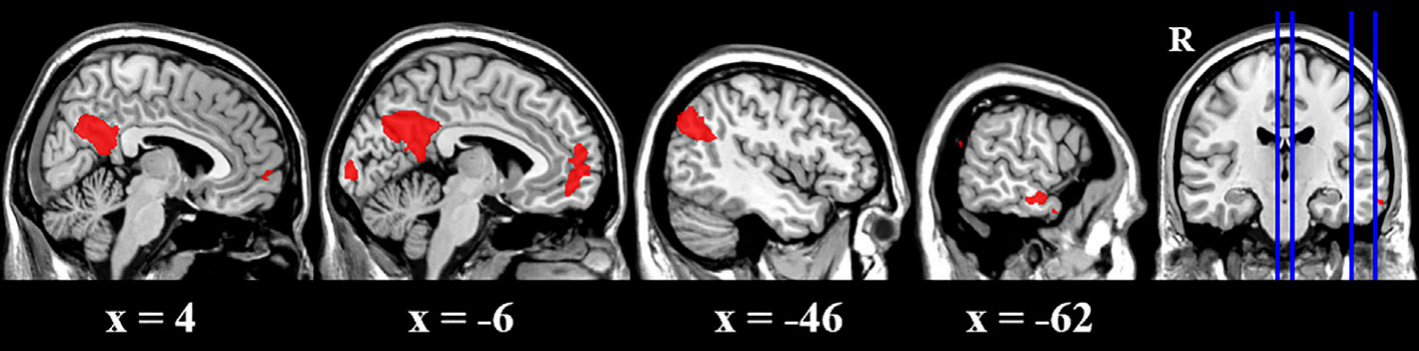
Brain activity difference between BQ images and control images in BQD group in the cue reactivity task. BQ images activated a large areas of brain than control images, including right ventromedial PFC, left posterior cingulate cortex (PCC), left lateral parietal lobe (LPL), left middle temporal gyrus and left visual cortex. However, in NCs group, these two categories of images showed no difference.

The cue reactivity task was a very simple task and both groups performed very well in this task (over 97% of correct ratio of detecting animals) (**Table 2**). There was no difference in terms of either correct ratio or reaction time between groups. To investigate the activity difference between BQ and control images, contrasts of BQ-control images were tested with one sample t-test for each group and two sample t-test for group difference. Results suggested that in BQD group, BQ images activated a large set of brain areas than control images did, including right ventromedial PFC, left posterior cingulate cortex (PCC), left lateral parietal lobe (LPL), left middle temporal gyrus and left visual cortex (**Table 3** and **Figure 1**). However, brain activity in response to these two categories of images showed no significant difference in NCs group as well as in comparison between BQD and NCs group.

**Table 2:** fMRI task behavioral data of different groups (M ± SD).

	BQD	NCs	Statistics
**Cue Reactivity Task**			
CR	0.99 ± 0.03	0.97 ± 0.05	*t*(68) = 1.77, *p* = 0.16
RT (ms)	609.5 ± 138.1	618.8 ± 144.3	*t*(68) = −0.25, *p* = 0.80
**Go/Nogo Task**			
BQ Go trial CR	0.92 ± 0.11	0.92 ± 0.12	*t*(68) = 0.74, *p* = 0.998
CN Go trial CR	0.85 ± 0.17	0.84 ± 0.13	*t*(68) = 0.30, *p* = 0.77
BQ Go trial RT (ms)	484.2 ± 36.6	486.8 ± 68.0	*t*(68) = −0.21, *p* = 0.84
CN Go trial RT (ms)	541.8 ± 62.5	551.7 ± 62.2	*t*(68) = −0.60, *p* = 0.55
BQ NoGo trial CR	0.85 ± 0.14	0.89 ± 0.07	*t*(68) = −1.25, *p* = 0.22
CN NoGo trial CR	0.84 ± 0.11	0.79 ± 0.15	*t*(68) = 1.41, *p* = 0.16

CR, correct ratio; RT, reaction time.

**Table 3:** fMRI activity of the cue reactivity task.

L/R	Brain Region	Voxels	MNI x	MNI y	MNI z	Z
**BQ-control contrast in BQD group**						
R	Ventromedial prefrontal cortex	535	0	58	−2	4.38
L	Posterior cingulate cortex	1793	−2	−52	30	6.87
L	Lateral parietal lobe	809	−50	−70	32	6.04
L	Middle temporal gyrus	164	−60	−8	−16	4.34
L	Visual cortex	210	−6	−98	2	5.00
**BQ-control contrast in NCs group**						
None						
**BQ-control contrast (BQD-NCs)**						
None						

### Go/Nogo Task

**Table 2** also summarized major behavioral measures for both fMRI Go/NoGo tasks, including the correct ratio for Go and NoGo trials as well as the reaction time for Go trials. There was no difference if we compared these variables across groups. The 2 (Task: Go vs. NoGo) *2 (Stimuli: BQ vs. Control images) *2 (Groups: BQD vs. NC) ANOVA was performed on the correct ratio. The results suggested that there was a significant main effect of task (F(1,67) = 4.87, p = 0.03; NoGo trials showed lower correct ratio than Go trials) and a significant main effect of stimuli (F(1,67) = 22.20, p < 0.001; BQ images showed higher correct ratio than control images). But there was no significant main effect of group or other interaction effects (all p > 0.05).

**Figure 2:**
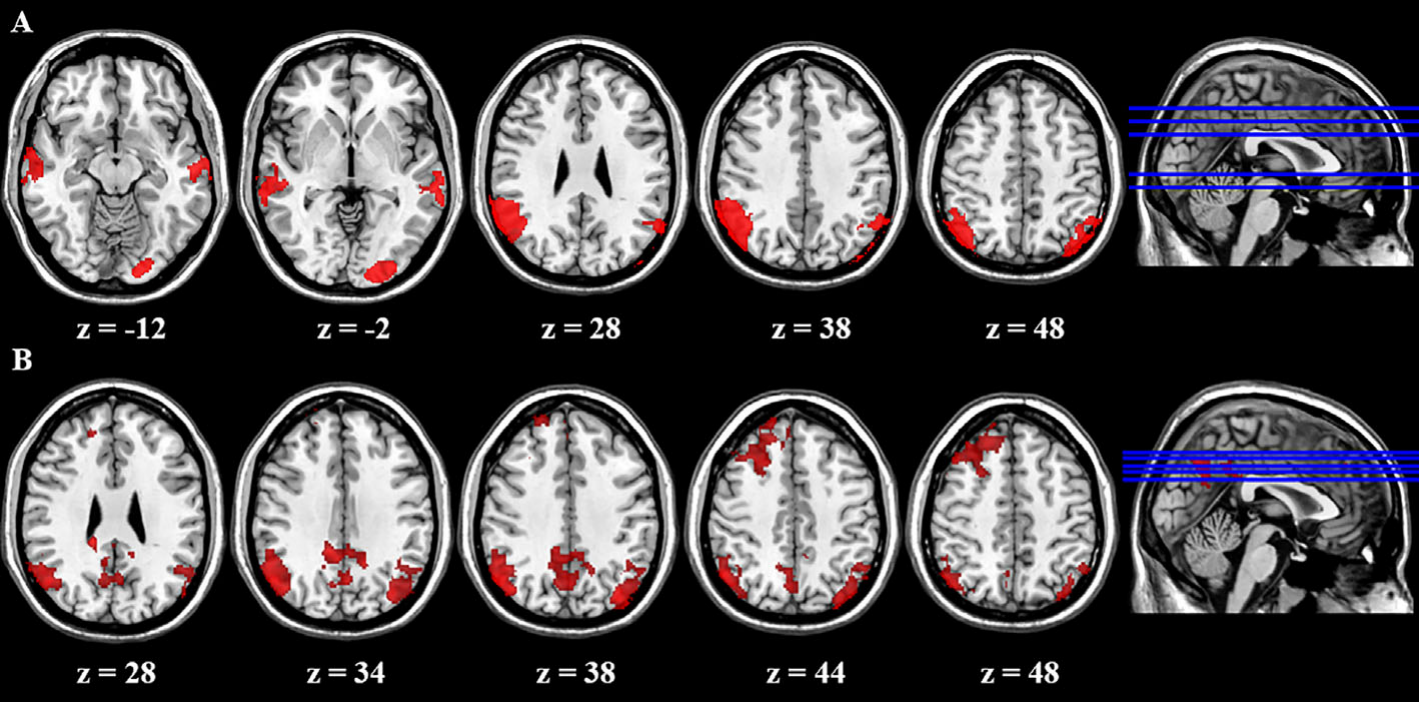
Brain activity difference between Nogo and Go trials in the Go/Nogo task. **(A)** Results suggested in BQD group, there was significant difference between NoGo and Go trials in the following regions: bilateral LPL, left lateral occipital cortex, and bilateral middle temporal gyrus. However, in NCs group, there was no difference when participants performed the Go/Nogo task. **(B)** Group comparison showed some brain regions showed higher activity in BQD than NCs group in right dorsolateral PFC, right PCC and bilateral LPL between NoGo and Go trials. However, there was no brain region with higher activity in the NCs than BQD group. The right hemisphere is displayed at the left side of the paper.

We first compared brain activity difference between BQ and control images in each group. Results suggested there was significant difference in BQD group between NoGo and Go trials in the following regions: bilateral LPL, left lateral occipital cortex, and bilateral middle temporal gyrus (**Table 4**). However, there was no difference in NCs group when participants performed the Go/NoGo task. Group comparison showed some brain regions with higher activity between NoGo than Go trials in BQD than NCs group, including right dorsolateral PFC, right PCC and bilateral LPL (**Table 4** and **Figure 2**). No regions showed higher activity in NCs group compared with BQD group.

**Table 4:** fMRI activity of the Go/NoGo task.

L/R	Brain Region	Voxels	MNI x	MNI y	MNI z	Z
**NoGo-Go trials in BQD group**						
R	Lateral parietal lobe	2567	54	−50	36	6.27
L	Lateral parietal lobe	1058	−58	−54	44	5.20
L	Lateral occipital cortex	851	−16	−96	−4	6.21
R	Middle temporal gyrus	803	64	−22	−8	5.46
L	Middle temporal gyrus	718	−64	−18	−6	5.37
**NoGo-Go trials in NCs group**						
None						
**NoGo-Go trials (BQD>NCs)**						
R	Dorsolateral prefrontal cortex	1205	32	32	52	4.40
R	Posterior cingulate cortex	1429	16	−36	28	4.95
R	Lateral parietal lobe	1606	52	−66	36	5.56
L	Lateral parietal lobe	1190	−36	−78	38	4.35
**NoGo-Go trials (NC>BQD)**						
None						

## Discussion

The present study aimed to investigate brain functional activity in PFC among subjects with BQD involved in the BQ-related cue reactivity task and Go/NoGo task. Behavioral results showed the BQD group showed deficits in the choice reaction time task but not in either the Stroop task or the working memory task. fMRI results of the cue task suggested that BQ images activated a large set of brain areas than control images did, including right ventromedial PFC, left PCC, left LPL, left middle temporal gyrus and left visual cortex when seeing BQ images compared with control images. In the Go/NoGo task, relative to NCs group, individuals with BQD showed higher activity in right dorsolateral PFC, right PCC and bilateral LPL between NoGo and Go trials. As hypothesized, individuals with BQD showed abnormal activation in PFC in both of these two tasks, which implies that functional abnormality of prefrontal area in BQ addiction.

These results highlighted functional alternation of PFC in BQD participants. Structural alteration in PFC has been widely reported in addiction, including cocaine (38–44), heroin (45–48), opiates (49), cannabis (50), nicotine (51, 52), alcohol (53, 54), ketamine (55), MDMA (56), methamphetamine (57), internet (58), and online games (59, 60). In the field of BQ addiction, our group study investigated the relationship between cortical thickness and executive function in chronic chewers with BQD, suggesting that BQD group had thinner cortex in bilateral PFC, and cortical thickness of the bilateral dorsolateral PFC mediated the correlation of betel-quid chewing and executive function (19). Consistent with decreased gray matter volumes in bilateral dorsolateral PFC and ventral medial PFC (22), results suggested that structural alteration in PFC in BQ users with BQD. Recently, several studies using functional connectivity approach to investigate resting-state brain feature in BQD users consistently suggested that impaired resting-state functional connectivity in BQ users occurred in prefrontal regions (61, 62). Our current study extends these prior findings by providing new evidence with task fMRI for abnormal functional activity in prefrontal regions in individuals with BQD.

It is worth noting that we observed abnormal activity in ventromedial PFC in cue reactivity task and dorsolateral PFC in Go/NoGo task. Prefrontal cortex is one of the most frequently areas implicated in drug addiction, which was involved in addicted subjects during intoxication, craving, bingeing and withdrawal (24). Altered prefrontal cortex activity has been reported in nicotine and cocaine addiction (63–65). Although activity among prefrontal cortex is highly integrated and flexible, a meta-analysis study suggests different region of prefrontal cortex is involved in different function (66). The dorsal PFC has been predominantly implicated in top-down control and meta-cognitive functions, the ventromedial PFC in emotion regulation (including conditioning and assigning incentive salience to drugs and drug-related cues), and the ventrolateral PFC and lateral OFC in automatic response tendencies and impulsivity (66). Consistent with previous studies in addition, our study provides novel evidence for the notion that abnormalities of ventromedial PFC in drugs and drug-related cues and dorsolateral PFC in top-down control in BQ dependence and further suggests that the dysfunction of functional activity in the ventromedial and dorsolateral PFC may contribute to impaired cue-induced craving and response inhibition in BQ dependence.

It should be noted that there were three limitations in current study. First, the study used an imbalanced sample. There were more individuals with BQD than normal participants. Second, this study only recruited male participants, so it should be cautious in generalizing the findings of this study to the females. Third, this study was done with outpatients, so there were inaccuracies regarding exact amount and histories of BQ use. Finally, although we performed a careful physical examination and obtained routine laboratory tests, all potential disease and confounding by other substances could not be totally excluded.

## Conclusion

Our study used neuroimaging approaches to assess the characteristic of brain activation with BQ specific cue reactivity and Go/NoGo tasks. The findings showed that individuals with BQD exhibited elevated responses in right ventromedial PFC, left PCC, left LPL, left middle temporal gyrus and left visual cortex when seeing BQ images compared with control images in the cue reactivity task. In the Go/NoGo task, relative to NCs group, individuals with BQD showed higher activity in right dorsolateral PFC, right PCC and bilateral LPL between NoGo and Go trials. Disrupted prefrontal function was consistently revealed in BQ cue induced craving and inhibitory control in individuals with BQD, which implies that treatments that target this region may help alleviate some symptoms of addictive BQ use.

## Data Availability Statement

The raw data supporting the conclusions of this article will be made available by the authors, without undue reservation.

## Ethics Statement

The studies involving human participants were reviewed and approved by the Institutional Review Board at Xiangya Hospital of Central South University of Changsha, Hunan Province, China. The patients/participants provided their written informed consent to participate in this study.

## Author Contributions

XZ conceived and designed the experiments. FY, SL, DW, and CJ conducted the experiments and collected data. ZZ and ZQ analyzed the results. LK and CZ wrote and revised the main manuscript text. All authors contributed to the article and approved the submitted version.

## Funding

This work was supported by research grants from the National Natural Science Foundation of China (grant number 61972460 to XZ and 61802443 to FY), Foundation for the Author of National Excellent Doctoral Dissertation of PR China (FANEDD) (grant number 201411 to XZ), Natural Science Foundation of Hunan Province (grant number 2019JJ20037 to XZ, 2020JJ4883 to LK, 2020JJ4923 to FY), Philosophy and Social Science Foundation of Hunan Province (grant number 17YBA426 to FY and 16YBA036 to XZ) and Youth Research of Xiangya Hospital, Central South University (grant number 2017Q19 to FY).

## Conflict of Interest

The authors declare that the research was conducted in the absence of any commercial or financial relationships that could be construed as a potential conflict of interest.
